# Comparative sequence analysis and mutagenesis of Ethylene Forming Enzyme (EFE) 2-oxoglutarate/Fe(II)-dependent dioxygenase homologs

**DOI:** 10.1186/1471-2091-15-22

**Published:** 2014-10-02

**Authors:** Nina Johansson, Karl O Persson, Christer Larsson, Joakim Norbeck

**Affiliations:** 1 Department of Chemical and Biological Engineering, Chalmers University of Technology, SE-412 96 Gothenburg, Sweden

## Abstract

**Background:**

Ethylene is one of the most used chemical monomers derived from non-renewable sources and we are investigating the possibility of producing it in yeast via the ethylene forming enzyme (EFE) from *Pseudomonas syringae*. To enable engineering strategies to improve the enzyme, it is necessary to identify the regions and amino acid residues involved in ethylene formation.

**Results:**

We identified the open reading frame for the EFE homolog in *Penicillium digitatum* and also showed its capability of mediating ethylene production in yeast. The sequence of the EFE homologs from *P.digitatum* and *P. syringae* was compared to that of the non-functional EFE-homolog from *Penicillium chrysogenum* and ten amino acids were found to correlate with ethylene production. Several of these amino acid residues were found to be important for ethylene production via point mutations in *P. syringae* EFE. The EFE homolog from *P. chrysogenum* was engineered at 10 amino acid residues to mimic the *P. syringae* EFE, but this did not confer ethylene producing capability.

Furthermore, we predicted the structure of EFE by homology to known structures of 2-oxoglutarate/Fe(II) dependent dioxygenases. Three of the amino acids correlating with ethylene production are located in the predicted 2-oxoglutarate binding domain. A protein domain specific for the EFE-class was shown to be essential for activity. Based on the structure and alanine substitutions, it is likely that amino acids (H189, D191 and H268) are responsible for binding the Fe(II) ligand.

**Conclusion:**

We provide further insight into the structure and function of the ethylene forming (EFE) - subclass of 2-oxoglutarate/Fe(II) dependent dioxygenases. We conclude that residues in addition to the 10 identified positions implicated in ethylene production by sequence comparison, are important for determining ethylene formation. We also demonstrate the use of an alternative EFE gene. The data from this study will provide the basis for directed protein engineering to enhance the ethylene production capability and properties of EFE.

## Background

Ethylene (C_2_H_4_) is a compound of considerable interest: First, it is an important plant hormone with strong effects on plant physiology and secondly, it is produced in high amounts for use in chemical synthesis (e.g. plastics). Understanding the enzymatic production of ethylene therefore has a dual biotechnological interest, both from the aspect of understanding and possibly using the effect on plants by microorganisms producing it, or by creating microorganisms which can convert biomass into ethylene for use in industry. In nature, ethylene is synthesized by three pathways, (1) the path from S-adenosylmethionine (ACC-pathway) is used in plants, (2) the KMBA pathway is used by several microorganisms and (3) a 2-oxoglutarate dependent pathway is used by several *Pseudomonas syringae* species and by *Penicillium digitatum*[1,2]. Pathway 3 has been shown to mediate the highest ethylene production, both naturally [2] and when expressed in heterologous hosts such as yeast [3,4]. This route is therefore the most promising for biotechnological production of ethylene [4], especially since expression of the plant system yielded very low ethylene production [5].

The biosynthesis of ethylene via the 2-oxoglutarate dependent ethylene forming enzyme (EFE) mainly from *Pseudomonas syringae* species but also from *Penicillium digitatum*, has been intensively studied, resulting in a proposed “dual-circuit” enzyme mechanism [6]. The overall stoichiometry of this reaction is that 2-oxoglutarate, oxygen and arginine are converted to carbon dioxide, ethylene, succinate, 1-pyrroline-5-carboxylic acid (P5C) and guanidine. However, this stoichiometry is the sum of two separate reactions: One (reaction 1) in which 2-oxoglutarate + oxygen produces ethylene and carbon dioxide, and another reaction (reaction 2) in which 2-oxoglutarate, arginine and oxygen is converted to succinate, P5C, carbon dioxide and guanidine.

Due to this duality in function the classification of the EFE is somewhat complex, it belongs to the 2-oxoglutarate/L-arginine monooxygenase/decarboxylase (succinate-forming) family (EC 1.14.11.34) which is also termed the Fe(II)/α-ketoglutarate-dependent hydroxylase family [7]. The hydroxylases are a large and rather heterogenous group of enzymes which in general employ 2-oxoglutarate and oxygen to hydroxylate a co-substrate. This co-substrate can be a wide range of molecules, e.g. proteins, nucleotides, lipids or other small chemicals, for review see [7], in the case of EFE the co-substrate is arginine. However, due to the ethylene forming reaction in which arginine is merely a co-factor rather than co-substrate, the EFE can also be classified as belonging to 2-oxoglutarate/Fe(II) dependent dioxygenases (EC 1.13.12.19) [6].

The aim of this study was to identify regions and amino acid residues of importance for ethylene production of EFE. We have achieved this through comparison of two ethylene producing homologs and one close homolog which does not produce ethylene. Protein structure was predicted and will, in combination with the sequence comparison, serve as a foundation for directed protein engineering to enhance the ethylene production capability of EFE.

## Results and discussion

### Cloning EFE from Penicillium digitatum

**Figure 1:**
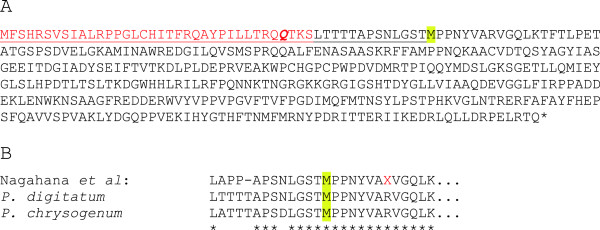
**Sequence of the EFE-protein from *P. digitatum*. (A)** Suggested revision of protein sequence of EFE from *P. digitatum*, isolate PHI26 (accession: EKV19239). In yellow shading is the present start codon of the protein EKV19239. Underlined sequence is translated from the 147 bp preceding the start codon. The sequence in red was not found in the purified EFE from *P.digitatum*. **(B)** Alignment of the sequences of *P. digitatum* and *P. chrysogenum* with the published N-terminal sequence of purified EFE from *P. digitatum* (indicated as: Nagahana et al.) [8]. The red X in the N-terminal sequencing is an R by homology, which fits well with the trouble of this amino acid in the chemistry of this method.

The ethylene forming activity of the 2-oxoglutarate dependent route is mediated by a single enzyme termed EFE, which has been biochemically characterized from isolates of *P. syringae* and from *P. digitatum*[8]. The EFE from *P. syringae* pv phaseolicola is especially well studied and has been shown to mediate ethylene formation when expressed in *S. cerevisiae, E. coli, T. reesii* and *P. putida*[9-12]. Unfortunately, the gene sequence of the enzyme from *P. digitatum* was not known, apart from the N-terminal 25 amino acids, characterized from a purified enzyme [8]. The recently published genome sequence of *P. digitatum*[13] allowed us to find a protein sequence with high homology to the *P. syringae* EFE. However, the previously published N-terminal was only partially found in this sequence and we therefore inspected the upstream codons in the DNA-sequence. This revealed that the predicted start codon is most likely not correct. When using the first alternative start codon, which is located 147 basepairs upstream, a similar sequence to the published N-terminal was found, preceded by a stretch of amino acids predicted by the MitoProtII-software (http://ihg.gsf.de/ihg/mitoprot.html) [14] to encode a mitochondrial signal peptide (Figure 1A). The probability for mitochondrial localization was 0.9491, with a predicted cleavage site after amino acid 33 (Bold/Italic in Figure 1A). This fits well, but not perfectly, with the N-terminal identified previously [8], indicating either that the protein is further processed after cleavage or that the predicted site of cleavage is inaccurate. In further support of our change in start codon choice for the *P. digitatum* EFE, a highly similar protein to the protein from *P. digitatum* is found in *Penicillium chrysogenum* (accession number: XP_002562422). The *P. chrysogenum* protein is predicted to use a start codon at a homologous position to that which we predict in *P. digitatum*. It is therefore likely that the *P. digitatum* EFE is a mitochondrially localized protein. This is somewhat surprising in view of the fact that *P. syringae* EFE was found to be inactive when expressed in mitochondria of *S. cerevisiae*[10]. Furthermore, there was a discrepancy in the first five amino acids of the *P. digitatum* EFE homolog and the sequence of the purified enzyme (Figure 1B). We do not know if this reflects a technical issue of the amino acid sequencing (in which the determination of arginine, proline and threonine can be difficult) or whether it reflects the fact that the sequences are derived from two different isolates of *P. digitatum*. This issue, together with the fact that sequence homology does not necessarily imply equal function, made it necessary to investigate whether the EFE-homologs from *Penicillium* species can mediate production of ethylene. To this end we cloned and expressed the EFE - homologs from *P. syringae*, *P. digitatum* and *P. chrysogenum* in *S. cerevisiae*. The EFE-genes from *Penicillium* were expressed without the putative mitochondrial targeting signal. When grown in batch cultures, both the *P. syringae* and *P. digitatum* enzymes mediated ethylene production, although the *P. digitatum* enzyme mediated only 68% of the ethylene production seen for the *P. syringae* enzyme (75 ± 14 vs 111 ± 12 μg/g_DW_ and hr, respectively; n = 2 for *P. digitatum* and n = 3 for *P. syringae*). The enzyme from *P. chrysogenum* produced no ethylene, despite its strong sequence similarity to the *P. digitatum* enzyme (data not shown).

### Sequence based identification of amino acids important for ethylene formation

**Figure 2:**
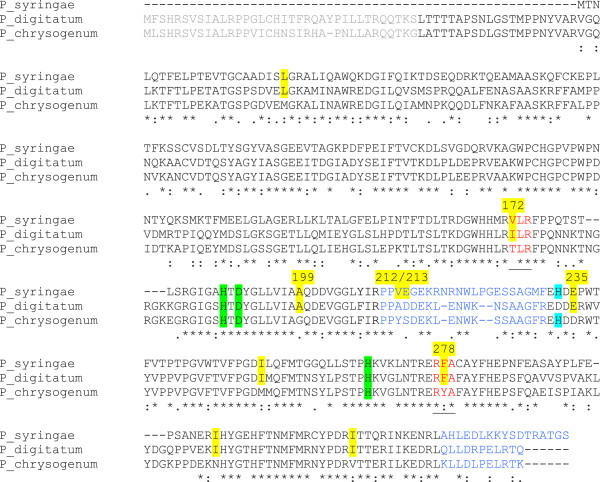
**ClustalOmega-alignment of the amino acid sequences of EFE-homologs from *P. syringae, P. digitatum *and *P. chrysogenum*.** Amino acids in yellow shading correlate with ethylene production. Amino acids in blue are not included in the predicted structural model. Green shaded amino acids are predicted to bind the Fe(II), the blue shaded histidines were previously suggested to bind Fe(II). Amino acids in red are predicted to bind 2-oxoglutarate and correspond to the EFE-site I [6]. Putative mitochondrial targeting sequences are marked in grey.

**Figure 3:**
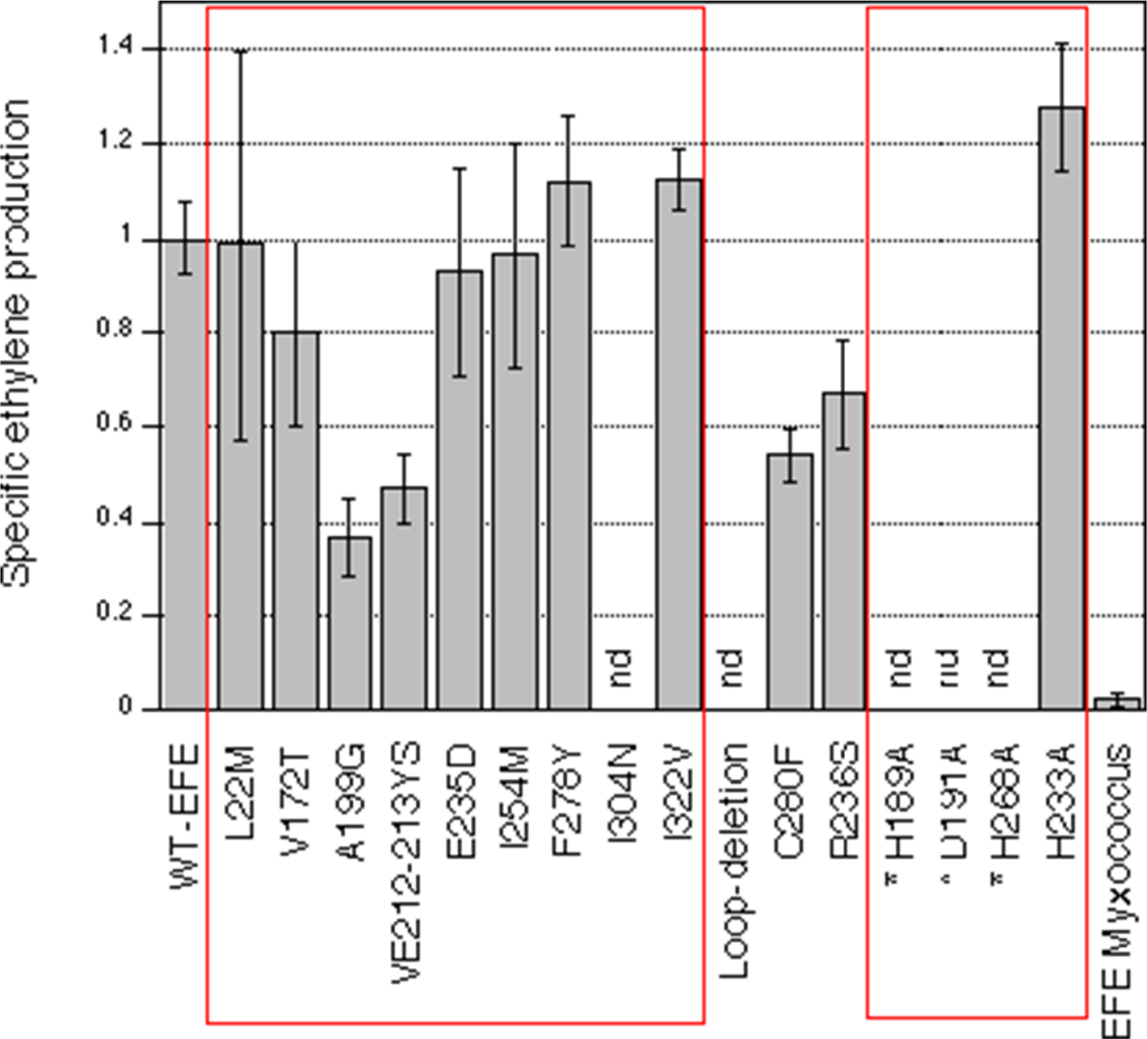
**Specific ethylene production of strains with plasmids containing mutations in EFE from *P. syringae*.** The production of the WT-EFE was set to 1. Standard deviation from at least 3 independent cultures. "nd" stands for not detectable. The large red rectangle indicates mutants in the 10 amino-acids correlating with ethylene production while the small red rectangle indicates mutations in amino acids putatively involved in iron binding.

Aligning of the sequences from the two ethylene producing homologs of EFE and the ethylene production negative homolog from *P. chrysogenum*, using ClustalOmega [15], subsequently allowed us to assign amino acids correlating with ethylene forming ability of the enzyme. Ten residues came out as promising targets, i.e. being conserved in *P. syringae* and *P. digitatum* but non-conserved in *P. chrysogenum* (Figure 2, yellow shading). In order to test the hypothesis that amino acids at these ten positions would be important for ethylene production, we made point mutations on all ten positions, i.e. L22M, V172T, A199G, V212Y/E213S, E235D, I254M, F278Y, I304N and I322V, thereby changing the amino acid to those found in the ethylene production negative EFE-homolog from *P. chrysogenum*. The A199G, I304N and V212Y/E213S mutants all showed a clear reduction of ethylene production, while L22M, V172T, E235D, I254M, F278Y and I322V did not cause a significant change in ethylene production (Figure 3).

C280 in *P. syringae* is close to the predicted 2-oxoglutarate binding motif (see below) and it is unusual in being a cystein. In most EFE homologs, including both the *Penicillium* sequences and the *Myxococcus* enzyme, a phenylalanine is found at the corresponding position, a non conservative substitution. Despite the fact that this amino acid was not implicated as crucial for ethylene production, we tested the effect of a C280F mutant for ethylene production. As can be seen in Figure 3, the mutation led to a reduction of ethylene production.

We also constructed a mutated version of the *P. chrysogenum* EFE-homolog, in which the 10 amino acids were exchanged for the amino acid found in *P. syringae*, since we expected that this would restore ethylene production. However, this hypothesis could be discarded since expression of this enzyme did not confer ethylene production (Data not shown). From these experiments we conclude that several of the 10 amino acids are indeed important for ethylene production, but that they are not sufficient in explaining why the two *Penicillium* enzymes differ in activity.

It is possible that the sequence signatures could be used to predict which EFE-homologs would produce ethylene and which would not. To test this we performed a BLAST search using the *P. syringae* EFE as a query. 102 hits with an E-value ≤1e-22 were scored. From this list of hits we selected enzymes which we believe will make ethylene, based on the conservation of motifs containing the 10 amino acids correlating with ethylene production. The top of the list consisted of homologs from different species of *Pseudomonas*, which was not surprising. A protein from *Myxococcus stipitatus* (YP 007362884.1) was found to contain conserved residues at all ten positions, with a BLAST E-value of 3e-164 and a 63% identity and a 98% query cover. (A ClustalOmega alignement of the *M. stipitatus* and *P. syringae* enzymes is provided as a supplement file (Additional file 1)). In comparison, the protein from *P. digitatum* had an E-value of 1e-122, an identity of 55% and a query cover of 95%. Since *Myxococcus* is a group of organisms evolutionarily far removed from *Pseudomonas*, and since no evidence of ethylene production from this class of bacteria exists, we decided to express the enzyme from *M. stipitatus* in yeast, from identical construct as the ones used for the other EFE-homologs. As can be seen from Figure 3, the *M. stipitatus* enzyme did indeed produce ethylene, albeit at low amounts. This is to our knowledge the first demonstration of a 2-oxoglutarate dependent EFE from a non-plant-pathogenic organism.

EFE-homologs from different isolates of *P. syringae* have been examined [16,17]. The most active EFE enzyme was that from the pathovar (pv) phaseolicola. The pathovars cannabina, sesami and glycinea displayed a roughly 50% reduced ethylene production while the enzyme from *P. syringae* pv pisi showed a 20-fold lower ethylene production. The pathovars cannabina, sesami and glycinea differ in sequence of EFE from that found in pv phaseolicola only in that R236 in phaseolicola is exchanged for an S, which is thus likely to explain the reduced ethylene production of these strains [17]. Also in our hands, an R236S mutation mediated a clear reduction of the ethylene production upon expression in yeast (Figure 3), further corroborating the importance of this site. From examination of the amino acid sequences it was found that the *P. syringae* pv pisi sequence lacks 13 amino acids on the C-terminal and differs in 29 amino acids over the aligned sequence [17]. 9 out of the 10 amino acid substitutions found to correlate with ethylene production in the comparison of two *Penicillium* EFE's and the EFE from *P. syringae* pv phaseolicola were conserved also in the *P. syringae* pv pisi-enzyme. However, the E235 position was exchanged for a D in the EFE from *P. syringae* pv pisi, which is the same as that found in the EFE from *P. chrysogenum*. However, although it is tempting to speculate that the strongly reduced ethylene production from the *P. syringae* pv pisi enzyme is due to the E235D-mutation, since the E235D mutant of the normal EFE did not show a major effect on ethylene production, it is instead likely that the reason for the low ethylene production is due to a combined effect of the other 29 amino acid substitutions and perhaps lack of the C-terminal.

### EFE-structure prediction

The ten amino acids correlating with ethylene production are distributed across the entire protein sequence of EFE. It was therefore of interest to investigate whether they would occur close to each other in the 3D-structure of the protein. However, the structure of EFE has not been determined for any of the three homologs, despite the fact that the protein can be produced at high levels in *E.coli*[18]. We have succesfully over-expressed EFE in *E. coli* and have attempted to determine the structure by both NMR and crystallography. However, for NMR it was found that labelling with deuterium causes protein precipitation (data not shown). A batch of highly purified EFE was also subjected to high throughput screening (SARomics, Lund, Sweden), but formation of crystals was not observed under any of the tested conditions. Therefore, to obtain indications of the EFE-structure, we submitted the amino acid sequences of *P. syringae*, *P. digitatum* and *P. chrysogenum* for structure prediction using the Phyre2-server (http://www.sbg.bio.ic.ac.uk/phyre2) [19]. All three sequences gave the same top hit, namely Leucoanthocyanidin dioxygenase from *Arabidopsis thaliana* (pdb-ID: 1gp6; http://www.uniprot.org/uniprot/Q96-323), henceforth termed LDOX. The structure prediction homology model for the EFE from *P. syringae* (and *P. digitatum* and *P. chrysogenum*) was downloaded and visualized using the Chimera software [20]. All three predicted models are found in the supplementary material with this paper (Additional files 2,3 and 4).

**Figure 4:**
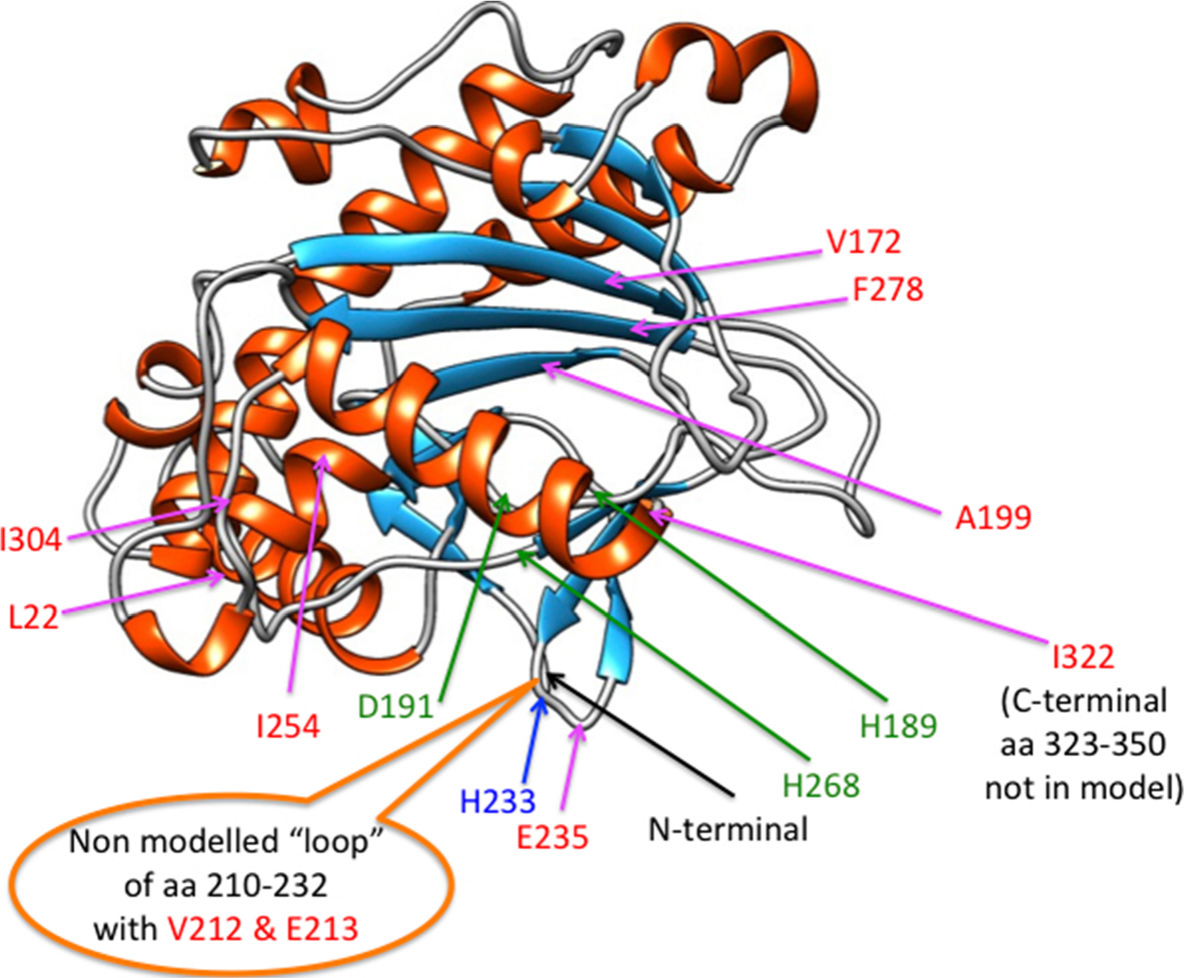
**Predicted structure of EFE from *P. syringae *pv phaseolicola, based on the structure of Leucoanthocyanidin dioxygenase from *Arabidopsis thaliana *generated by the Phyre2 server.** Amino acids marked in red correlate with ethylene production, amino acids in green are involved in binding Fe(II), Histidine 233 is marked in blue and the orange callout-bubble corresponds to the non-modelled "loop".

Studies of the LDOX enzyme have indicated that the regions corresponding to positions 172-174 and 277-279 in the *P. syringae* EFE are involved in binding of the 2-oxoglutarate [21], these regions are shown in red in Figure 2. The involvement of an arginine (at position corresponding to R277 in *P. syringae* EFE) in binding the C-5 of 2-oxoglutarate has previously been suggested [7]. Interestingly, both of these regions contain one residue correlating with ethylene formation, V172 is isoleucine (I) in *P. digitatum* and threonine (T) in *P. chrysogenum*, the former being a conservative replacement and the latter constituting a shift from hydrophobic to hydrophilic group. Similarly, the phenylalanine (F) at position 278 is substituted for a more hydrophilic tyrosine (Y) residue in the ethylene-negative EFE from *P. chrysogenum*. The predicted structure of *P. syringae* EFE from Phyre2 results (Figure 4) show that in addition to the previously mentioned V172 and F278, the A199 which also correlates with ethylene production, is located close to the others, in the beta-sheet region thought to be involved in 2-oxoglutarate binding. The A199G mutation is somewhat conservative, but is likely to influence structure. The amino acids 172-174 and 277-279 are predicted to comprise at least part of the 2-oxoglutarate binding site (Site I) as previously proposed in the dual circuit reaction model of EFE [6]. Since the spatial alignement of this site I has been proposed to determine whether the ethylene formation (reaction 1) or the arginine/2-oxoglutarate breakdown (reaction 2) occurs, it was interesting that 3 of the 10 amino acids (V172, F278 and A199) occurred in this domain. However, only the A199G mutant led to a reduction of ethylene production (Figure 3). The I304N is a non-conservative mutation and was also found to display the greatest effect on ethylene production (Figure 3). The strong effect of a I304N-mutation could be due to alteration of the position of the predicted C-terminal alpha-helix which we find it likely will change the structure of the essential iron-binding site (Figure 4).

From the structure modelling and sequence alignment it was evident that EFE from *Pseudomnoas syringae* and from the *Penicillium* EFE-homologs have an additional stretch of amino acids (corresponding to amino acids 210-232 in *P. syringae* EFE) which is not present in the non-ethylene forming structural homolog from *Arabidopsis* (Figures 2 and  4). This region is predicted to be unstructured. However, 2 positions in this non-modelled "loop" correlate with ethylene production, i.e. position 212 is a small hydrophobic residue (valine (V) or an alanine (A)) and position 213 is an acidic amino acid (aspartate (D) or glutamate (E)) in the EFE homologs mediating ethylene production. In contrast, the EFE from *P. chrysogenum* has a tyrosine (Y) followed by a serine (S), both polar residues, at the corresponding positions. The function and structure of this "loop" is at present not known, but deletion of the corresponding amino acids from the *P. syringae* enzyme leads to loss of function ("loop deletion", Figure 3). Furthermore, a VE212-213YS mutation displayed a reduced ethylene production (Figure 3).

From the predicted structure it is easy to envision that the non-conservative mutations in the 2-oxoglutarate binding site would affect ethylene production. The other substitutions would be predicted to mediate a more subtle change in the enzyme structure, possibly leading to a "stretching" of the active site previously suggested to favour the ethylene forming reaction [6]. However, the Phyre2-based structure prediction of the normal syringae EFE and a sequence with 10 substitutions to the amino acids found in the chrysogenum EFE showed no major difference.

### Iron binding site of EFE

Previously, based on point mutations of histidines to glutamines in EFE from *P. syringae*[22], it was suggested that H189 and H233 would be the main iron binding residues. However, the histidine 233 is not conserved in *P. digitatum*, where it is an aspartate, making it unlikely that H233 would have this role. Furthermore, we find that a H233A mutation is fully active (Figure 3). This is in strong contrast to the previous finding that a H233Q mutant is inactive for ethylene production [22], but we cannot at present provide an explanation for this. Also, from the structural and sequence homology to LDOX and other members of this enzyme class, we believe that histidines 189 and 268 together with aspartate 191 are involved in iron binding, which has also been proposed previously [3,7]. It is also evident from the structure prediction (Figure 4) that the histidine 233 would be located on the outer side of the protein, further making its involvement in iron binding unlikely. The fact that alanine substitutions of H189, H268 and D191 all result in complete abolition of ethylene production (Figure 3) strongly supports their role in binding the Fe(II) co-factor.

## Conclusions

We have corrected the predicted sequence and cloned the EFE from *P. digitatum*, demonstrating that it is somewhat less efficient than EFE from *P. syringae* in terms of productivity upon expression in yeast. An EFE-homolog from *M. stipitatus* was even less efficient for ethylene production while the EFE-homolog from *P. chrysogenum* was inactive.

By amino acid homology, we have furthermore identified amino acid residues and domains important for ethylene production, although it is clear that other residues are important for the EFE-activity. We also suggest a structure for the EFE class of 2-oxoglutarate/Fe(II) dependent dioxygenases. We anticipate that a more detailed comparison of several heterologously expressed EFE-enzymes with varying ethylene forming efficiency, in combination with the suggested protein structures, will enable improvement of EFE-function, and will thus be valuable for further development of ethylene production in microorganisms.

Why are homologs of EFE common, while comparatively few of the organisms carrying these homologs seem to make ethylene? It is possible that the role of EFE-homologs in most organisms is to make P5C from arginine by using 2-oxoglutarate as a co-substrate with formation of succinate. This would adhere to the more "normal" reaction mechanism for this enzyme class [7]. The formation of ethylene from the co-substrate 2-oxoglutarate could have been selected as beneficial for certain plant pathogens, such as *P. digitatum* and *P. syringae*. EFE seems to be important for the ability of *P. syringae* to proliferate in plants [23]. However, it is still unclear which role ethylene formation plays in the pathogenesis, e.g. by *P. syringae* and *P. digitatum*, since plants also produce ethylene as part of their stress response, including infection by pathogens [24,25]. A detailed understanding of the enzyme EFE itself could therefore contribute to elucidating the role of ethylene production in plant pathogenes.

## Methods

### Cloning of EFE-homologs

The coding regions for the EFE-homologs from *P. digitatum*, *P. chrysogenum* and *M. stipitatus* were ordered as synthetic, codon-optimized genes (GeneScript, Piscataway, NJ). The *P. chrysogenum* and *M. stipitatus* EFE homologs were expressed via integration of a *TDH3*-promotor regulated construct in the genome (1000 bp downstream of the *DAK2* gene) as performed previously [26,27]. The *P. digitatum* and *P. syringae* EFE homologs were expressed in a multicopy plasmid (pYX212), for the *P. digitatum* EFE using the sequence following the predicted mitochondrial targetting sequence (red amino acids in Figure 1), preceded by a methionine-codon.

The plasmid constructs bearing homologs of EFE were transformed into S. cerevisiae strain CEN.PK 113_5D (MATa MAL2-8^c^ SUC2 ura3-52) while the strain CEN.PK 113_7D (*MAT*a *MAL2-8*^c^*SUC2*) was used for the genomic integrations.

### Growth of yeast and detection of ethylene

Throughout this study a minimal mineral media [28], with 10 g glucose L^−1^ was employed. Double amounts of trace metals compared to the original version were used, as well as somewhat higher KH_2_PO_4_ and MgSO_4_ levels, 3.5 g L^−1^ and 0.75 g L^−1^ respectively. The vitamin solution was prepared according to the original published version. 7,5 g L^−1^ of ammonium sulfate was used as nitrogen source throughout the study.

For the experiments in Figure 3, cells were grown in 250 ml Erlenmeyer shake flasks with 50 ml liquid cell culture. Minimal mineral media was inoculated with ON cultures to OD_600_ of 0.4. The cell cultures were grown at 30°C, 200 rpm until OD_600_ in-between 1-2 was reached. Custom made rubber caps with syringe needles were used to trap the gases produced inside the flask. The cultures were plugged with caps 30 minutes ahead of sampling. Gas samples were extracted from the shake flasks using 50 ml syringes. The gas samples were subsequently injected to the loop of a HP5890II GC-FID (Hewlett-Packard, USA) with a HayeSep Q 80/100 porous packed column (Grace, USA). The GC column was heated to a constant 65°C, injection temperature 70°C and detection temperature 125°C. The ethylene detected from the bioreactor was compared to an ethylene standard with a known concentration to determine the amount. The samples were analyzed using the ChromNav version (1.14A) software (JASCO, Japan).

For the evaluation of ethylene production from the *Penicillium* strains, 2 L aerobic batch cultivations were performed in 3.5 L Belach (Belach Bioteknik AB, Skogås, Sweden) bioreactors in the above described minimal media. Overnight shake flask cultures were used to inoculate the bioreactor to a starting OD_600_ of 0.1. In the bioreactor temperature and air flow were held constant at 30°C and 1 L min^−1^ respectively. Agitation was maintained around 800 rpm and the pH was kept at 5.0 using 1M NaOH and 1M HCl. Off-gas and dissolved oxygen were monitored using the Bleach gas-analyzer module CP460 and regulatory module CP400 respectively. Ethylene was determined directly in the off gas by connecting the off-gas via a pump with over pressure release to the GC-FID (as described above).

## Competing interests

The authors declare no competing interests.

## Authors’ contributions

NJ and KP performed all experimental work and participated in the writing of the manuscript. CL and JN wrote the manuscript. All authors read and approved the final manuscript.

## Supplementary Material

Additional file 1 **Alignment of EFE from *P. syringae *** **with EFE from *M. stipitatus.*** Showing position of the 10 amino acids correlating with ethylene production. Click here for file

Additional file 2 **Protein model of EFE from *P. syringae.***
Click here for file

Additional file 3 **Protein model of EFE from *P. digitatum.***
Click here for file

Additional file 4 **Protein model of EFE from *P. chrysogenum.***
Click here for file
