# Supplementary material for: Comparative sequence analysis and mutagenesis of Ethylene Forming Enzyme (EFE) 2-oxoglutarate/Fe(II)-dependent dioxygenase homologs
Source: BMC Biochem. 2014 Oct 2;15:22. doi: 10.1186/1471-2091-15-22 (PMC4202696; doi:10.1186/1471-2091-15-22)
Supplement: Additional file 1 — Alignment of EFE from P. syringae with EFE from M. stipitatus. Showing position of the 10 amino acids correlating with ethylene production. [file 1471-2091-15-22-S1.pdf]

```
EFE_M_stipitatus      MIELETFQLPQSVSGREADIALGLTMVRAWRRDGIFQVRMSPAQAEKSQRAFELSRHFFR     60
EFE_P_syringae        MTNLQTFELPTEVTGCAADISLGRALIQAWQKDGIFQIKTDSEQDRKTQEAMAASKQFCK     60
                      * :*:**:** .*:*  ***:** ::::**::*****:: .  * .*:*.*:  *::* :

EFE_M_stipitatus      QSLETKARCVSDLTYSGYIASGQELTASEADLSEVFTVCRDVPLTDPRVQSKWPCHGPGP     120
EFE_P_syringae        EPLTFKSSCVSDLTYSGYVASGEEVTAGKPDFPEIFTVCKDLSVGDQRVKAGWPCHGPVP     120
                      : *  *: **********:***:*:**.: *: *:****:*: : * **:: ****** *

EFE_M_stipitatus      WPDESWRQGMQAHAEELGSVGERLLRLIALGLGLDIDALTTLTHDGWHHMRVLRFPARSP     180
EFE_P_syringae        WPNNTYQKSMKTFMEELGLAGERLLKLTALGFELPINTFTDLTRDGWHHMRVLRFPPQTS     180
                      **::::::.*::. **** .*****:* ***: * *:::* **:************ :: 

EFE_M_stipitatus      TTTRGIGAHTDYGLLVIAAQDDVGGLYVRPPVEGEKRPRNWLPHESSAGMYEHDEPWTYV     240
EFE_P_syringae        TLSRGIGAHTDYGLLVIAAQDDVGGLYIRPPVEGEKRNRNWLPGESSAGMFEHDEPWTFV     240
                      * :************************:********* ***** ******:*******:*

EFE_M_stipitatus      KPVPGVLTVFPGDILQFLTRGYLLSTPHKVVLNTRERFALAYFHEPQFEACVRPLSAPTR     300
EFE_P_syringae        TPTPGVWTVFPGDILQFMTGGQLLSTPHKVKLNTRERFACAYFHEPNFEASAYPLFEPSA     300
                      .*.*** **********:* * ******** ******** ******:***.. **  *: 

EFE_M_stipitatus      DEYIHYGTHFTNMFMRSYPDRVTTQRILDESRLTTLSWLRQEAVLRTAPLEAVPLQRAAG     360
EFE_P_syringae        NERIHYGEHFTNMFMRCYPDRITTQRINKENRLAHLEDLKKYSDTRATGS----------     350
                      :* **** ********.****:***** .*.**: *. *:: :  *::
```
